# A Woman with Asthma and Peripheral Ground-Glass Opacities

**DOI:** 10.1155/2019/9051381

**Published:** 2019-02-12

**Authors:** Chia-Yu Chiu, Michael David, Aya Musa, Mohmed Gora, Manmeet Kaur Gujral

**Affiliations:** ^1^Department of Internal Medicine, Lincoln Medical Center, NY, USA; ^2^Department of Internal Medicine, Section of pulmonary, Lincoln Medical Center, NY, USA

## Abstract

Eosinophilic lung disease is a heterogeneous group of disorders that reveal eosinophil involved lung tissue often in patients with asthma or atopy. Classification and diagnostic criteria of eosinophilic lung disease are not well-established; however, peripheral ground-glass opacity is typical on chest computed tomography. Another etiology of this same radiographic finding reported in the literature is silicone embolism syndrome. Here, we present a 43-year-old female with poorly controlled severe persistent asthma presenting with difficulty breathing. Computed tomography showed peripherally dominant ground-glass opacity. Peripheral blood, bronchoalveolar lavage fluid analysis, and transbronchial biopsy did not find eosinophilia. Serial bronchoalveolar lavage of the demonstrated increasingly blood-tinged fluid. The patient required mechanical ventilation upon admission. After further questioning the patient revealed that she had frequently received injectable cosmetics at non-licensed establishments. Initially, due to past medical history, presentation, and radiographic findings, eosinophilic pneumonia was suspected. However, after a review of the patient's social history and risk factors, silicone embolisms syndrome became a likely diagnosis. The patient had good clinical response to high dose steroid therapy.

## 1. Introduction

Eosinophilic lung disease describes a diverse group of diffuse, parenchymal pulmonary disorders by the presence of pulmonary tissue eosinophilia and/or peripheral blood eosinophilia. Eosinophilic lung disease can be classified as primary (unknown cause) or secondary (known cause) [1, 2]. Diagnosis of eosinophilic lung diseases can be based on any of the following: peripheral blood eosinophilia (absolute eosinophil count ≥ 500 eosinophils/microL) with abnormalities on pulmonary imaging, eosinophils in bronchoalveolar lavage (BAL) fluid, tissue eosinophilia confirmed in transbronchial or open lung biopsies [1]. Secondary eosinophilic lung disease is associated with drugs, toxins, helminthic infections, fungal infections, connective tissue disorders, and neoplastic processes. Primary eosinophilic lung disease can be simplified to idiopathic acute eosinophilic pneumonia and chronic eosinophilic pneumonia. Idiopathic acute eosinophilic pneumonia presents as a rapidly progressive hypoxemic respiratory failure with possible hypersensitivity reaction to unidentified inhaled antigen. Although there is no consensus on diagnostic criteria in idiopathic eosinophilic pneumonia, experts have proposed five clinical criteria for patients with possible idiopathic acute eosinophilic pneumonia: acute onset of less than 1 month, bilateral diffuse infiltration on pulmonary imaging, hypoxemic respiratory failure, bronchoalveolar lavage eosinophilia exceeding 25% or eosinophilic pneumonia at lung biopsy, and exclusion of other etiologies of secondary eosinophilic pneumonia [3]. Initially, the eosinophilic fraction may not be elevated in peripheral blood in many patients. IgE levels are high in the majority of patients but this is not included in the diagnostic criteria [1]. On imaging, bilateral reticular ground-glass opacity is common but consolidation can also be found. Prompt response to steroids without relapse is a unique feature of idiopathic acute eosinophilic pulmonary disease [4].

Chronic eosinophilic pneumonia is a rare disorder more frequently occurring to females with atopic features. Asthma is present in more than 50% of cases. Symptoms gradually progress over 1 month. Peripheral eosinophilia is present in 90% of patients [1, 2]. Eosinophil accumulation in pulmonary alveoli is estimated by pathology specimen. Diagnosis of chronic eosinophilic pneumonia is based on clinical presentation, peripheral eosinophilia, BAL eosinophilia, and biopsy specimen [5]. On imaging, chronic eosinophilic pneumonia is characterized by the presence of homogeneous peripheral airspace consolidations, where bilateral is more common than unilateral. Irreversible fibrosis and air-bronchograms have been reported in some patients [4]. Unlike acute eosinophilic pneumonia, chronic eosinophilic pneumonia needs long-term steroid use to prevent relapse [5].

Silicone Embolism Syndrome (SES) was first reported by Chastre in 1983 in The New England Journal of Medicine and now is frequently reported elsewhere in the literature [6]. SES is an entity associated with recent injection of cosmetic silicone. Patients develop respiratory distress and nonspecific flu-like or gastrointestinal symptoms. CT often reveals bilateral peripheral ground glass consolidation [6–10]. BAL can reveal hemorrhage and biopsy may show increased macrophages or lipoid material. One case series found alveolar hemorrhage in 64% of patients [8]. The pathogenesis is either direct accidental intravenous injection or dissemination of silicone by neutrophils, macrophages and other immune cells. Visualization of vascular filling defects and lobar infarctions are infrequently seen. The result of the dissemination of silicone is an immune response which can be acute or chronic.

## 2. Case Presentation

A 43-year-old female with past medical history of asthma and bipolar disorder presented to our emergency room with progressive chest pain and shortness of breath for 3 days. She had been prescribed alprazolam, lamotrigine, prednisone, and albuterol for more than 10 years but was not compliant to her medication. Further questioning revealed that she took oral prednisone in recent days because of shortness of breath. She described non-exertional pressure like pain over the middle of her chest. The pain was 10/10, constant, without radiation, localized to the retrosternal area, and aggravated by deep breathing. Exercise tolerance was reported as less than 1 block due to shortness of breath. Patient denied fever, wheezing, fatigue, chill, nausea, vomiting, diarrhea, constipation, joint pain, or rash. Patient lived in Maryland but recently traveled to Miami two weeks prior to presentation and only arrived in New York one week before presentation. Upon further question, the patient's brother revealed that she had been going to unlicensed establishments and receiving silicone injections in her buttocks. She did not reveal the exact time she had these injections but stated she had come to New York to have the procedure.

**Figure 1:**
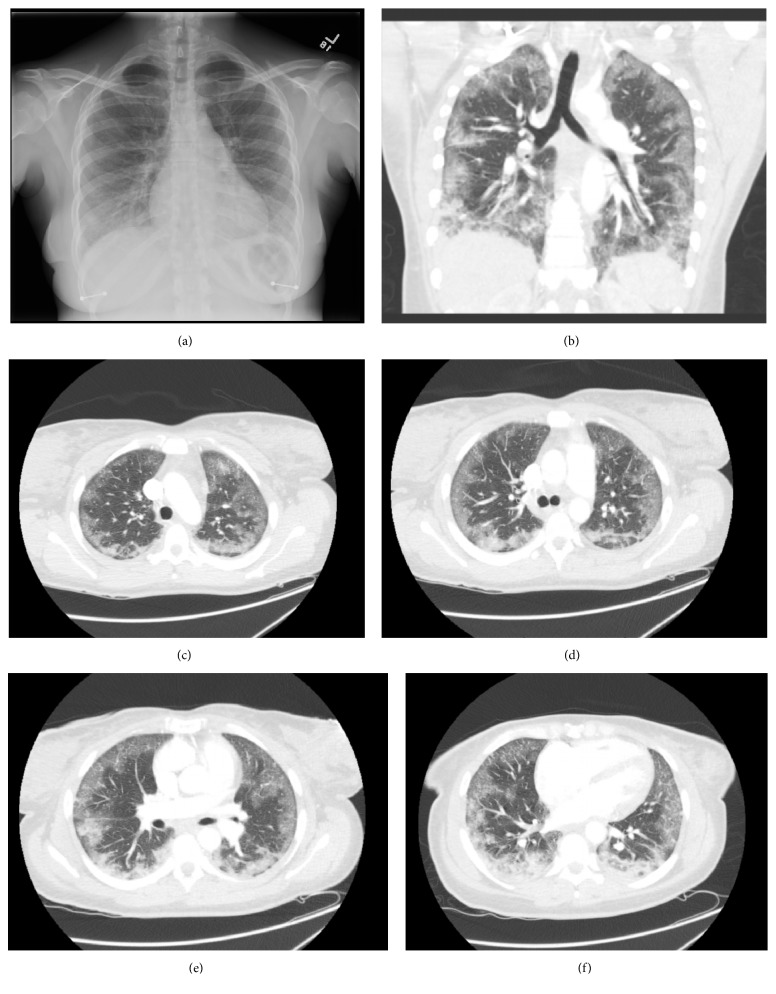
(a) Chest X-ray PA view shows increase attenuated over peripheral lung field without significant consolidation or nodularity. (b–f) Computed tomography of chest shows extensive, symmetric alveolar opacities with interspersed foci of interlobular septal thickening, subpleural sparing, and scattered ground-glass nodularity over the bilaterally periphery lung field, with perihilar sparing. No mediastinal, hilar lymphadenopathy or pleural effusion.

On physical examination, the patient was afebrile. Her heart rate and blood pressure were normal, and her respiratory rate was 20 breaths/minute. Arterial oxygen saturation was 93% on ambient air. On auscultation, crackles were heard over bilateral lung fields, without wheezing. Cardiovascular, abdominal, neurological, musculoskeletal, and skin examinations were unremarkable. A chest radiograph demonstrated increase bilateral peripheral lung field opacities (Figure 1(a)). Computed tomographic (CT) imaging of the chest showed peripheral predominant ground-glass opacities. No bronchiectasis or fibrosis was noted (Figures 1(b)–1(f)). After admission to general medicine, her respiratory rate increased to 29 breaths/min with labored breathing and accessory muscle use. Patient received rapid-sequence endotracheal intubation for severe hypoxia. She was treated with a lung protective strategy (tidal volume of 6cc/kg of ideal body weight; plateau pressure less than 30 cm H2O). Broad-spectrum antibiotics, including ceftriaxone and azithromycin, was instituted. High-dose methylprednisolone was also initiated and gradually tapered to oral prednisone. Further laboratory investigation, including renal and liver function tests, serum antinuclear antibody, anti-neutrophil cytoplasmic antibodies (Myeloperoxidase antibody/Proteinase-3 antibody), IgG levels specific to Aspergillus (Aspergillus niger/Aspergillus flavus/Aspergillus fumigatus), stool ova/parasite, syphilis testing, influenza A and B by PCR, respiratory syncytial virus by PCR, legionella urine antigen, and procalcitonin were negative. Urine toxicology was negative for benzodiazepines, cannabinoids, opioid, cocaine, and phencyclidine. Total IgE is 149 KU/L (reference ≤100 kU/L). We performed bronchoscopy with BAL and transbronchial biopsies on day 8 of admission. Serial BAL of the right lower lobe demonstrated increasingly blood-tinged fluid from tube 1 to tube 5. Cell count performed on the BAL fluid showed red blood cell 24,484/mm3, white blood cell 89/mm3, segment 65%, lymphocyte 28%, monocyte 1%, and mesothelial cell 6%. Grocott-Gomori methenamine silver stain of the fluid was negative for* P. jirovecii*. Bacterial, viral, and fungal cultures were all negative. Tests for acid-fast bacilli and ova and parasites were also negative. Transbronchial biopsies showed benign bronchial alveolar tissue with inflammation; no eosinophils were found. The day after bronchoscopy and extubation, the patient left against medical advice in stable condition.

## 3. Discussion

The patient's clinical presentation was initially worked up to rule out common causes of shortness of breath and chest pain. Entities like asthma exacerbation, pulmonary embolism, and coronary artery disease were ruled out, and concurrent imaging studies revealed a new diagnostic pathway. The peripheral ground-glass CT findings offer a narrow differential suggestive of chronic eosinophilic pneumonia, organizing pneumonia, vasculitis, and aspiration. This patient did not have an endemic area travel history and lack of gastrointestinal symptom makes diagnosis helminth infection less likely. The patient did not have extrapulmonary manifestations such us skin rash, joint pain, or uveitis. Lack of serology makes connective tissue disease less likely in this patient. Infectious disease workup was also all negative. Due to history of asthma, eosinophilic disease secondary to asthma or infection was initially suspected based on clinical and radiographic findings.

BAL revealed blood consistent with diffuse alveolar hemorrhage. Acute eosinophilic pneumonia, chronic eosinophilic pneumonia, and SES have all been associated with diffuse alveolar hemorrhage [10–12]. Bilateral ground-glass opacity on chest CT narrows down the differential diagnosis to eosinophilic lung disease, organizing pneumonia, sarcoidosis, and SES [13]. Peripheral ground-glass opacity was first described by Gaensler and Carrington as a chest X-ray pattern in chronic eosinophilic pneumonia in 1977 [14]. In that report, 53 of 81 (65%) cases of chronic eosinophilic pneumonia had peripheral opacities. However, further studies revealed eosinophilic lung diseases to share the same radiographic pattern [1–3, 15]. Therefore, the reverse batwing sign expands the differential to all eosinophilic lung [16]. Peripheral consolidation and peripheral ground-glass appearance are both non-specific for eosinophilic lung disease, although one study by Mochimaru reveals peripheral consolidation is found more frequently in chronic eosinophilic pneumonia and peripheral ground-glass opacity is found more frequently in acute eosinophilic pneumonia [4]. Distribution of opacity and laterality cannot reliably identify or distinguish these two [4, 9].

This patient with asthma had peripheral ground-glass opacities, anchoring our clinical thinking to the diagnosis of acute eosinophilic pneumonia. Elevated IgE level can be present in eosinophilic lung disease but is not diagnostic [1, 3, 16]. However, this patient did not show eosinophilia on peripheral blood, bronchoalveolar lavage, or tissue biopsy. Though in patients with intermittent steroid use eosinophilia may not be seen, this missing factor led us to question this diagnosis. The history to silicone injections induced us to inquire about this specific exposure. Interestingly, SES shares many of the same characteristics as eosinophilic pneumonia. Bilateral, peripheral opacities on imaging and diffuse alveolar hemorrhage can be seen in both [17]. Unfortunately, the tissue biopsy did not reveal a definite diagnosis. Steroids are the recommended treatment of eosinophilic pneumonia and SES so it was not possible to distinguish them based on clinical response.

## 4. Conclusion

A variety of diseases are associated with diffuse alveolar hemorrhage and ground-glass appearance. Diffuse alveolar hemorrhage can occur secondary to any injury or inflammation of lung and has been reported in both eosinophilic lung disease and silicone-related lung injury. Radiographic characteristics of eosinophilic lung disease are ambiguous and must be correlated with past medical history, clinical presentation, and laboratory specimen. Complicating the matter is that acute eosinophilic pneumonia may not show eosinophilia by either peripheral blood or bronchoalveolar lavage, especially after recent prednisone use. Elevated IgE level may hint towards eosinophilic lung disease. In specific cases where these factors are not present and the diagnosis is still dubious, it is obviously wise to expand the differential. An uncommon though pertinent risk factor is silicone exposure. SES may arise similarly to eosinophilic pneumonia and should be considered when this exposure is present. Further study is still needed to understand the exact pathomechanism of SES and delineate optimal diagnosis and treatment.
